# Strategic considerations for advancing research in intradural extramedullary cervical schwannomas: a call for a multicenter registry

**DOI:** 10.1016/j.clinsp.2026.101130

**Published:** 2026-10-05

**Authors:** Ilker Sengul, Demet Sengul

**Affiliations:** a Thyroidology Unit, Giresun University Faculty of Medicine, Giresun, Turkey; b Division of Endocrine Surgery, Giresun University Faculty of Medicine, Giresun, Turkey; c Department of General Surgery, Giresun University Faculty of Medicine, Giresun, Turkey; d Department of Pathology, Giresun University Faculty of Medicine, Giresun, Turkey

Dear Editor,

The management of cervical intradural extramedullary schwannomas represents a significant challenge due to their anatomical proximity to the spinal cord and their tendency to mimic more common neurological disorders. We read with interest the case report by Romero and colleagues,^1^ which underscores the diagnostic difficulty inherent in these rare lesions.

A critical observation in recent clinical reports is the prolonged diagnostic latency. As seen in the case of Romero et al.^1^ the initial clinical suspicion often veers toward conditions such as amyotrophic lateral sclerosis, ALS, due to the manifestation of quadriparesis and fasciculations. This diagnostic ambiguity necessitates a rigorous differential framework, particularly when myelopathic signs progress rapidly after a period of indolence.

While the efficacy of multilevel laminectomies and total excision is well-documented, the clinical priority lies in optimizing surgical timing and monitoring through standardized data. Due to their rarity, intradural extramedullary cervical schwannomas require further investigation to improve our understanding of this condition.

There is a pressing need for the establishment of a multicenter registry dedicated to rare spinal intradural lesions. Such a database would allow for a more nuanced understanding of long-term outcomes and the development of a standardized diagnostic algorithm. Implementing such a registry involves significant challenges, including the harmonization of data across different surgical centers and the requirement for consistent funding to maintain data integrity.

In conclusion, the clinical community must shift its focus toward large-scale data collaboration through a multicenter registry to ensure that surgical interventions are guided by robust epidemiological evidence. We thank the authors for their elegant report on schwannomas, 2,3 which remain a crucial global health issue.

## Data availability statement

The datasets used and/or analyzed during this study are available from the corresponding author upon reasonable request.

## Authors’ contributions

Ilker Sengul: Conceptualization, writing – original draft.

Demet Sengul: Conceptualization, writing – review & editing.

## Funding

None declared.

## Conflicts of interest

The authors declare no conflicts of interest.

## References

[1] Romero N., Aransibia L., Gutierrez Moyano A. (2025). On the clinical and surgical interpretation of cervical schwannoma: in response to considerations. Medicina.

